# Superpixel-Based PSO Algorithms for Color Image Quantization

**DOI:** 10.3390/s23031108

**Published:** 2023-01-18

**Authors:** Mariusz Frackiewicz, Henryk Palus, Daniel Prandzioch

**Affiliations:** Department of Data Science and Engineering, Silesian University of Technology, Akademicka 16, 44-100 Gliwice, Poland

**Keywords:** color image quantization, clustering, particle swarm optimization, individual difference evolution, superpixel, image quality, computation rate

## Abstract

Nature-inspired artificial intelligence algorithms have been applied to color image quantization (CIQ) for some time. Among these algorithms, the particle swarm optimization algorithm (PSO-CIQ) and its numerous modifications are important in CIQ. In this article, the usefulness of such a modification, labeled IDE-PSO-CIQ and additionally using the idea of individual difference evolution based on the emotional states of particles, is tested. The superiority of this algorithm over the PSO-CIQ algorithm was demonstrated using a set of quality indices based on pixels, patches, and superpixels. Furthermore, both algorithms studied were applied to superpixel versions of quantized images, creating color palettes in much less time. A heuristic method was proposed to select the number of superpixels, depending on the size of the palette. The effectiveness of the proposed algorithms was experimentally verified on a set of benchmark color images. The results obtained from the computational experiments indicate a multiple reduction in computation time for the superpixel methods while maintaining the high quality of the output quantized images, slightly inferior to that obtained with the pixel methods.

## 1. Introduction

Color image quantization (CIQ) is a transformation of a true color image (usually 8 bits for each RGB component) into an image with some colors, e.g., 8, 16, 32, 64, 128, and 256, which make up the color palette. Such a significant reduction in the number of colors in an image can enable faster, further image processing for such tasks as image compression [1], image segmentation [2], content-based image retrieval [3], etc. It is important to keep maximal similarity between the original image and the quantized image. Therefore, color image quantization can be considered a useful preprocessing operation.Although there are different CIQ methods, they consist of two main operations, which are the design of the color palette and the mapping of the pixels of the original image to the colors of the palette.

A few decades of development of the CIQ subject matter have resulted in several different approaches. The first methods can be called splitting methods. They split the solid color into smaller and smaller boxes, whose representatives (e.g., mean colors) are included in the final color palette. A good example of these is the classic method of median cut [4]. Splitting methods are fast, but quantized images often differ significantly from the original images. The second category of methods utilizes pixel clustering algorithms such as K-means (KM) [5] and fuzzy C-means (FCM) [6,7]. The clustering of pixels in a color image based on the unsupervised classification of millions of pixels compares their color similarities. The clustering methods used in CIQ provide high-quality quantized images, but their high computational complexity is the reason for the long quantization time. Additionally, their results depend on the initial conditions.

At the end of the last century, attempts were made to apply genetic [8] and evolutionary [9] algorithms for color quantization. Since 2005, work on applying swarm-based algorithms to color quantization has taken off. To solve this image preprocessing task, various metaheuristics have been used to mimic the behavior of groups of individuals, such as particles [10], ants [11], bacteria [12], frogs [13], fishes [14], bees [15], fireflies [16], wolves [17], etc. Recently, researchers have started using hybrid methods, that is, methods composed of several simpler algorithms, for color quantization. An example of this approach is the ant algorithm combined with the firefly algorithm [18] and a combination of the particle swarm optimization algorithm and the ant algorithm [19]. The goal of further work in the CIQ area will be methods that generate high-quality quantized images in a short time.

The aforementioned swarm and evolutionary algorithms belong to a large group of stochastic methods used in both optimization and simulation tasks [20]. Swarm intelligence algorithms were developed as a result of observations of nature in which different herds cooperate and exchange information between individuals. This leads to intelligent collective behavior that allows us to solve difficult problems. One of the first such algorithms was proposed in 1995 by J. Kennedy and R. Eberhart, the particle swarm optimization algorithm (PSO) [21]. In the PSO algorithm, each particle represents a candidate solution to a given optimization problem. The change in the position of a particle and, therefore, the change in the potential solution in each iteration, depends on the best known position of a given particle and the best known position of a particle from the entire swarm. One of the main advantages of swarm algorithms with respect to traditional optimization algorithms is that they start their operation from a population of potential solutions, rather than from a single point.

The PSO algorithm was first applied to the task of quantizing color images in 2005 [10]. The proposed PSO-CIQ algorithm combines the PSO algorithm with the K-means clustering algorithm. Before the values of the function are calculated, the particles are randomly selected and their positions are then refined by several iterations of the K-means algorithm.

For many years, PSO modifications have focused mainly on the selection of parameters (e.g., the number of particles in a swarm, acceleration coefficients, inertia weight, etc.), the modification of the neighborhood topology (e.g., grid, circle, star, and random), and the development of hybrid algorithms combining PSO with other metaheuristics (e.g., evolutionary algorithms). However, competition between particles was generally ignored. Among the many modifications of the recently developed PSO algorithm, we should note the version that also uses the evolution of individual differences (IDE-PSO) [22]. The authors of this algorithm introduced an evolutionary approach using individual differences. The algorithm assigns a coefficient called emotional status to each particle to determine individual differences, divides the whole swarm into three groups, and chooses an evolutionary method for each particle depending on its emotional status.

One way to reduce the CIQ time may be to preprocess the image by segmenting the image into superpixels and setting the color palette based on superpixels rather than pixels [23]. An image has fewer superpixels than pixels, so algorithms that operate on superpixels can run faster than pixel-based algorithms. The most popular method for generating superpixels is SLIC (simple linear iterative clustering) [24]. This algorithm uses the K-means method and classifies pixels based on color and their location. The distance between pixel colors is computed in the perceptually uniform CIELab color space. Each superpixel is assigned a mean color derived from the colors of its pixels. The number of superpixels in an image, their size, or their compactness depends on the type of superpixel generator. Of the characteristics listed here, color plays the most important role in the CIQ process, both in the pixel and superpixel versions. Splitting an image into superpixels, if performed quickly, can improve the efficiency of such image processing operations as CIQ or image segmentation. In the work [23], the authors proposed superpixel versions of three classic CIQ algorithms: median cut, K-means, and FCM. This approach allowed multiple decreases in computation time (15–30 times) with a minimal reduction in image quality.

In addition to the shorter execution time of CIQ operations, operation with limited computer resources was achieved. Image quality was evaluated using multiple indices (PSNR, FSIMc, DSCSI, HPSI, and SPSIM). An important parameter of superpixel CIQ algorithms is the number of superpixels into which the image was decomposed. It depends only on the number of quantization levels.

This study investigated the suitability of the IDE-PSO algorithm for CIQ tasks and the possibility of speeding up calculations due to the use of the superpixel approach (SP-IDE-PSO). The remainder of this paper is organized as follows. Section 2 presents the PSO-CIQ algorithm, its modification named IDE-PSO-CIQ, and our proposed superpixel versions of both methods. In Section 3, the experimental results and their discussion are shown. Finally, Section 4 concludes the article.

## 2. Materials and Methods

Swarm algorithms are a family of global optimization techniques inspired by the collective behavior of individuals (particles) that form a set of candidates to solve an optimization problem. A swarm of particles moves through the parameter space according to trajectories that depend on the best results of their own and those of their neighbors. Swarm particles perform rather simple tasks without central control. These particles actively seek the minimum of the objective function, work together in a topology-dependent manner, and swarm and memorize the previously obtained good results.

### 2.1. PSO-CIQ [10]

Applying the PSO algorithm to the CIQ task should define the space through which the particles will move. The first such application was carried out by Mahamed Omran in his work [10]. Each particle *i*-th $X_{i}$ can be described as

(1) $$X_{i}=\left\{x_{i,1},x_{i,2},\ldots ,x_{i,k},\ldots ,x_{i,K}\right\},$$

where *K*—the number of colors in the color palette of the particle. The components of the particle are three-dimensional color vectors:

(2) $$x_{i,k}=\left[R_{i,k},G_{i,k},B_{i,k}\right].$$

Therefore, each particle in the swarm represents a different color palette, while the quality of each particle is evaluated on the basis of a quality index such as MSE. The pseudocode of the PSO-CIQ algorithm is shown below (Algorithm 1).
**Algorithm 1** PSO-CIQ [10].1:Initialize each particle by choosing K colors from the RGB color space of the image and take them as initial pBest values.2:Calculate the values of the objective function f(.) for each particle and find the initial gBest value.3:**while** Stop condition of the algorithm **do**4:      **for** For each *i*-th particle **do**5:            Apply several iterations of the K-means algorithm with probability pkmeans6:            Update the particle velocity (Equation (3)) and position (Equation (4))7:            Calculate the value of the objective function f(.) for the particle8:            Update the personal best solution for the particle (Equation (5))9:            Update the best global solution (Equation (6))10:    **end for**11:**end while**

The initialization of the starting positions for each particle in the swarm (Step 1) is performed by randomly selecting *K* colors from the color space of the original image. Initially, these values are regarded as the personal best positions of the particles (pBest). For all particles, we find the global best solution, which minimizes the chosen objective function f(.), in our case the MSE.

The main loop of the algorithm is then executed (Step 3). The algorithm is run and executed until a stop condition is met. The stop condition in this case is to reach the maximum number of iterations tmax. At the beginning of each iteration (step 5), a random number is determined by a continuous uniform distribution of the interval [0;1]. If this number has a value less than the value of the pkmeans parameter, then on the color palette represented by the particle in question there are several iterations of the K-means algorithm. This procedure reduces the image quantization error that results from the random start of the particle swarm. It also makes it possible to obtain much better final quantization results, both in terms of minimizing the quality index and improving the visual aspects of the image after quantization. In the next step, the particle velocity and position are updated:

(3) $$V_{i}^{t}=wV_{i}^{t-1}+c_{1}r_{1}\left(pBest_{i}^{t-1}-X_{i}^{t-1}\right)+c_{2}r_{2}\left(gBest^{t-1}-X_{i}^{t-1}\right),$$

(4) $$X_{i}^{t}=X_{i}^{t-1}+V_{i}^{t}.$$

In Step 7, the value of the objective function for the *i*-th particle is determined. However, to determine the value of the objective function, one must first determine a new image from the color palette that the particle in question represents in a given iteration. To do this, calculate the distance between each pixel of the original image and each color in the color palette. Furthermore, the pixels are assigned to the colors of the palette for which the calculated distance has the lowest value. After the mapping of the image pixels to palette colors is performed, the MSE quality index of the image after quantization is determined. The value of this index is the value of the objective function of the particle. In the following steps (8,9), the personal and global best solutions are updated:

(5) $$if\;\;\;\;f\left(X_{i}^{t}\right)\leq f\left(pBest_{i}^{t}\right)\;\;\;\;then\;\;\;\;pBest_{i}^{t}=X_{i}^{t},$$

(6) $$if\;\;\;\;f\left(X_{i}^{t}\right)\leq f\left(gBest^{t}\right)\;\;\;\;then\;\;\;\;gBest^{t}=X_{i}^{t}.$$

The global best solution, and therefore the best color palette to date, is the solution represented by that particle for which the value of the objective function takes the lowest value.

After each time they are determined, a mechanism is used to prevent particles from “escaping” from the optimized space. In the case of CIQ problems, this space is the RGB color space with components in the range from 0 to 255. Thus, when a color from the palette represented by one of the particles takes a value outside this range, it is then limited to the limiting value of this range. A similar mechanism is also applied to the velocity of particles. In this case, the acceptable range of particle velocities is assumed to be $\left[-V_{max};V_{max}\right]$.

The following values were assumed for the parameters of the PSO-CIQ algorithm:

- NP—swarm size;
- pkmeans=0.1—probability of applying the K-means algorithm;
- $t_{max}=50$—total number of iterations;
- $w_{max}=0.72$, $w_{min}=0.4$—inertia weight coefficients;
- $c_{1}=1.49$—exploration coefficient;
- $c_{2}=1.49$—exploitation coefficient;
- $V_{max}=255$—maximum velocity value.

To strengthen the initial exploration phase and the final phase of exploitation, the following inertia weight coefficient was used, the value of which decreases linearly with successive iterations. Therefore, it was assumed that the initial value of the inertia weight coefficient was $w_{max}=0.72$, while the final value of this coefficient decreased to $w_{min}=0.4$. When using the K-means algorithm, 10 iterations were assumed.

### 2.2. IDE-PSO-CIQ

In 2017, a new modification of the PSO algorithm was proposed [22], which introduced an evolutionary mechanism into the algorithm that uses individual differences between particles. The IDE-PSO algorithm assigns to each particle a competition factor eX, called an emotional state, to quantify individual differences between particles. In this algorithm, based on the emotional state of each particle, the swarm is divided into three subgroups: weak, normal, and good. Depending on the particle’s affiliation to a given subgroup, its behavior changes, and thus its movement in the optimized multidimensional space (Algorithm 2).
**Algorithm 2** IDE-PSO-CIQ.1:Initialize each particle by choosing K colors from the RGB color space of the image.2:Initialize each particle velocity at random and emotional states.3:Calculate the objective function for the first generation of particles.4:**while** Stop condition of the algorithm **do**5:      Group the particles with respect to their emotional state eX6:      **for** For each *i*-th particle **do**7:            **while** Stagnation *Stag* counter value reached **do**8:                 Update the position of the particle according to the mechanism corresponding to the its emotional state9:                  **if** If the position is within the limits of the search space **then**10:                     Then accept the new position and break the stagnation loop11:                **else**12:                     Reject the new position and increment the value of the stagnation counter13:                **end if**14:           **end while**15:           Apply several iterations of the K-means algorithm with probability *pkmeans*16:     **end for**17:     Evaluate the fitness of the current swarm18:     Update particles’ historical best position and the global best position19:     Update emotional status eXi of particles depending on the change in their change of fitness20:**end while**

The emotional state $eX_{i}$ of the *i*-th particle is initialized based on a random number *r* with a continuous uniform distribution in the interval [−0.1;0.1] (Equation (7)):

(7) $$eX_{i}^{0}=r.$$

On the contrary, the emotional state in each iteration is determined by the value of the objective function of all particles. If the actual value of the objective function is better than the value of the previous iteration, then the emotional state of a particle grows by the value of $\Delta^{+}$ (Equation (8)). Similarly, if the value of the objective function of a particle is less than the value of the previous iteration, its emotional state decreases by the value $\Delta^{-}$ (Equation (9)):

(8) $$\Delta^{+}=\frac{\left(f\left(X_{i}^{t-1}\right)-f\left(X_{i}^{t}\right)\right)\cdot \left(f\left(X_{i}^{t}\right)-f\left(gb^{t}\right)\right)}{{\left(f\left(gw^{t}\right)-f\left(gb^{t}\right)\right)}^{2}},$$

(9) $$\Delta^{-}=\frac{\left(f\left(X_{i}^{t}\right)-f\left(X_{i}^{t-1}\right)\right)\cdot \left(f\left(gw^{t}\right)-f\left(X_{i}^{t}\right)\right)}{{\left(f\left(gw^{t}\right)-f\left(gb^{t}\right)\right)}^{2}},$$

where the symbols are defined as follows:

- f(.)—the value of the objective function for a given solution;
- $X_{i}^{t}$—the position of the *i*-th particle in a given iteration;
- $X_{i}^{t-1}$—the position of the *i*-th particle in the previous iteration;
- $gb^{t}$—the best global position among all particles in a given iteration;
- $gw^{t}$—the worst global position among all particles in a given iteration.

Furthermore, the closer a particle is to the best global solution, the smaller the value by which its emotional state changes.

In the IDE-PSO algorithm, three emotional states of the particles are defined: weak, normal, and good. Accordingly, the particles in each iteration are dynamically assigned to three subgroups based on the value of their emotional state. To split the particles into three groups, the particles are segregated according to their emotional state eX in such a way that the particle with the worst emotional state is the first in line. Two coefficients, $m_{1}$ and $m_{2}$, whose values are in the range [0;1], are used as limits to assign particles to the three groups of the algorithm. Thus, we have a swarm of NP particles, and the allocation to the three emotional groups is performed as follows, where $ind_{i}$ is the index of the *i*-th particle in the set of particles grouped by their emotional state:

- $ind_{i}\leq m_{1}\times NP$ — particles with a weak emotional state;
- $m_{1}\times NP<ind_{i}\leq m_{2}\times NP$ — particles with a normal emotional state;
- $m_{2}\times NP<ind_{i}$ — particles with a good emotional state.

Particles with a poor emotional state tend to move in the wrong direction or are more likely to stop at the local minimum of the optimized space. Therefore, the mechanism of moving weak particles leads the particles to abandon their current position and learn from the best particles in the swarm. The way in which weak particles change their position depends on a random number *r* with a continuous uniform distribution in the interval [0;1]. A given particle is iterated relative to each *j*-th dimension. If the value of the random number *r* is less than the number 0.5, then the dimension *j*-th of the particle is set as the dimension *j*-th of the best global position (Equation (10)):

(10) $$X_{i}^{t}\left(j\right)=gb^{t-1}\left(j\right).$$

Alternatively, if the value of the random number *r* is greater than or equal to 0.5, then the *j*-th dimension of the particle is taken as a random number from the interval belonging to the optimized space:

(11) $$X_{i}^{t}\left(j\right)=l+\left(u-l\right)rand\left[-1;1\right],$$

where *l* and *u* are the upper and lower limits of the optimized space dimension, respectively, and rand[−1;1] is a random number in the range [−1;1].

Particles with a normal emotional state determine their new position in both, based on their own previous position and the best global position among the swarm. The formula to determine the velocity (Equation (12)) and position (Equation (13)) of these particles is the same as for the original version of the PSO algorithm:

(12) $$V_{i}^{t}\left(j\right)=\omega V_{i}^{t-1}+c_{1}r_{1}\left(pBest_{i}^{t-1}-X_{i}^{t-1}\right)+c_{2}r_{2}\left(gBest^{t-1}-X_{i}^{t-1}\right),$$

(13) $$X_{i}^{t}=X_{i}^{t-1}+V_{i}^{t},$$

where the symbols are defined as follows:

- $V_{i}^{t}$—the velocity of the *i*-th particle in a given iteration;
- $V_{i}^{t-1}$—the velocity of the *i*-th particle in the previous iteration;
- $pBest_{i}^{t-1}$—the best known position of the *i*-th particle;
- $gBest^{t-1}$—the best known position from the entire swarm;
- $c_{1},c_{2}$—the coefficient of exploration and exploitation;
- $r_{1},r_{2}$—random numbers with uniform continuous distribution from the interval [0;1];
- ω—the coefficient of inertia weight.

In a situation where a particle has a good emotional state, it stays confident because its value of the objective function has improved over the previous few iterations. Therefore, its next position is determined only on the basis of its past, regardless of the positions of other particles in the swarm. The mechanism for updating the velocity and position of these particles is presented as follows:

(14) $$V_{i}^{t}\left(j\right)=\omega V_{i}^{t-1}\left(j\right)+0.2\omega \left(u-l\right)rand\left[-1;1\right],$$

(15) $$X_{i}^{t}=X_{i}^{t-1}+V_{i}^{t}.$$

The second term in Equation (14) is added to the previous value of the velocity of the particle to improve its exploration ability.

The IDE-PSO algorithm additionally uses the Stag stagnation parameter. This parameter was introduced to prevent the particles from escaping from the optimized space. If a particle in a given iteration finds itself outside the search area, its newly determined position is abandoned, and a new position is recalculated. Each time a particle is found outside the search area, the counter of the stagnation parameter is incremented. If the value of the counter reaches the value of the Stag parameter, then the particle remains in the same place.

### 2.3. Superpixel Versions of PSO-CIQ and IDE-PSO-CIQ Algorithms

**Figure 1:**
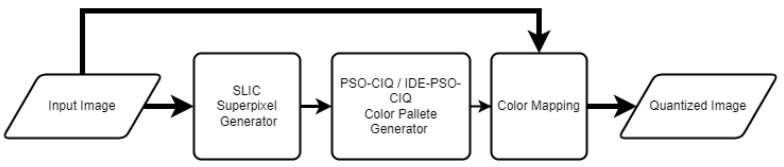
General idea of SP versions of the PSO-CIQ and IDE-PSO-CIQ methods.

The proposed superpixel CIQ methods, presented in Figure 1, use the original image as input to the superpixel generator (in our case, SLIC) and as input to the color pixel mapping. The generation of the color palette, the most complicated stage of the CIQ method, uses only the superpixel image. This proposed approach should significantly decrease the computation time and shorten the CIQ operation.

We used three algorithms to determine color palettes. To complete the operation, the color of each pixel within the original image is mapped to its nearest color from the palette. Introducing superpixels into the CIQ algorithm would decrease computational complexity while producing only a small reduce in image quality. We propose modified superpixel versions of the PSO algorithms: SP-PSO-CIQ and SP-IDE-PSO-CIQ. We compare these SP algorithms with their original pixel versions. We consider the dependence between the number of superpixels generated $N_{SP}$ and the quality of the final results. $N_{SP}$ should not be less than the number of colors *k* in the palette. To specify $N_{SP}$, we propose the following empirical formula:

(16) $$N_{SP}=k+SP\_Ratio\cdot k,$$

where SP_Ratio∈{2,4,8,16}.

**Figure 2:**
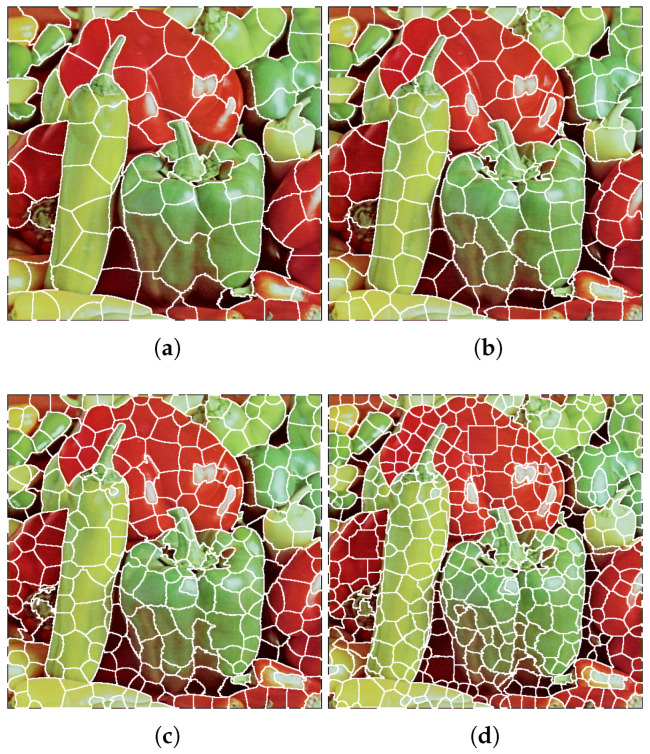
Superpixel decomposition for image *Peppers* with K=32. (**a**) Image splitting into 96 superpixels ($N_{SP}=2$). (**b**) Image splitting into 160 superpixels ($N_{SP}=4$). (**c**) Image splitting into 288 superpixels ($N_{SP}=8$). (**d**) Image splitting into 544 superpixels ($N_{SP}=16$).

The application of this formula to divide the image into superpixels is illustrated in Figure 2. Using a quantization level of *k* = 32, four images split into 96, 160, 288, and 544 superpixels were generated from the input image. With these tools, we can produce superpixel images, perform CIQ, and objectively evaluate image quality after quantization, allowing us to evaluate and assess superpixel CIQ methods.

The computational complexity of the PSO algorithm is linearly dependent on the size of the swarm and the number of input data. For the PSO-CIQ algorithm applied to digital images, the computational complexity is linearly dependent on the size of the swarm and the number of pixels in the image [10]. A simple analysis of the IDE-PSO-CIQ algorithm shows that its complexity is also O(n). The main factor affecting the computation time is the number of pixels, and therefore reducing this number by introducing superpixels significantly affects the computation time, making it possible to obtain high values of computation rates.

### 2.4. Evaluation of Image Quality

Methods for evaluating image quality mainly refer to images with specific distortions. Comparatively, not much research has been conducted on the evaluation of image quality after CIQ-caused changes [25].

To verify image quality, we used ten different image quality indices, including classic mean squared error (MSE), peak signal-to-noise ratio (PSNR), and mean color error (DE). Each of above indices could be considered equally suitable for application to CIQ image changes. However, in recent research, indices have been proposed that are better correlated with the human perception.

For this work, we also chose the following modern quality indices: the color feature similarity index (FSIM) [26], the sparse feature fidelity index [27], the improved color-image-difference (ICID) index [28], the directional statistics color similarity index (DSCSI) [29], the Haar wavelet perceptual similarity index (HPSI) [30], and the mean deviation similarity index (MDSIS) [31]. The usability of the latter indices within the CIQ field has already been proven [32]. We also used the superpixel similarity (SPSIM) index proposed for application to superpixel segmentation results [33,34]. Most of these quality indices require maximizing values; only MSE, DE, ICID, and MDSIS are minimized.

## 3. Experiments, Results, and Discussion

All research was performed on a desktop computer with a CPU AMD Ryzen Threadripper 3960X at 3.80 GHz and 128 GB of RAM. The algorithms were implemented in the Matlab R2021b.

### 3.1. PSO-CIQ *vs.* IDE-PSO-CIQ

**Figure 3:**
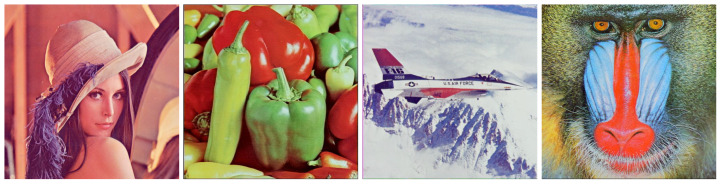
Selected benchmark color images.

The paper [10] shows, for four benchmark color images: *Lenna*, *Peppers*, *Jet*, and *Mandrill*, that the PSO-CIQ algorithm generally achieved better results than the compared SOM and GCMA algorithms. Each benchmark image has a spatial resolution of 512 × 512 pixels—Figure 3. Table 1 of this paper has been supplemented with a column containing the results obtained for the proposed IDE-PSO-CIQ algorithm. In ten of the twelve cases studied, the IDE-PSO-CIQ algorithm achieved lower MSE values than PSO-CIQ.

In order not to be limited to the classic MSE index, we conducted similar studies on the same images for nine other pixel-based (PSNE, DE), patch-based (FSIM, SFF, ICID, DSCSI, HPSI, MDSIS), and superpixel-based (SPSIM) indices. The values of the indices are positively correlated with the image quality. The results of such a multi-index evaluation are included in Table 2. The bold values indicate the best of these for each image in the three palette sizes used. As in the previous experiment, the results of the IDE-PSO-CIQ algorithm dominated the PSO-CIQ results (94 of 108 comparisons in favor of the IDE version).

### 3.2. Superpixel Versions of Both Algorithms

**Figure 4:**
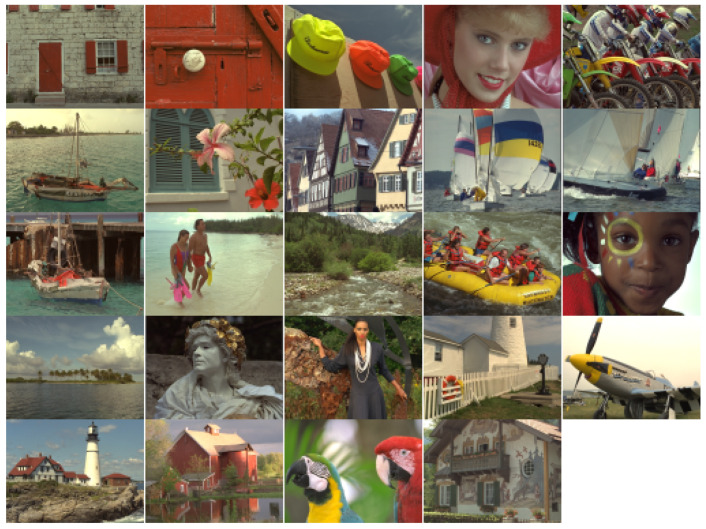
Set of Kodak test images.

The proposed superpixel versions of the PSO-CIQ and IDE-PSO-CIQ algorithms were tested on 24 test images from the Kodak test images (Figure 4) for 3 palette sizes (16, 64, and 256).

The average results obtained for both pixel and superpixel versions at different swarm sizes (5 and 20) are given in Table 3, Table 4 and Table 5 and the best ones are in bold. The quality of quantization is higher when a larger swarm size is used. From the tables discussed, it can be seen that the transition to superpixels results in a slight loss of quantized image quality. The use of a superpixel approach preserves the advantage of IDE-PSO-CIQ over PSO-CIQ. Generally, the IDE-PSO-CIQ algorithm achieved much better color quantization results in terms of the values of the quality indices. Two indices were chosen to evaluate the loss of quality in the quantized image: HPSI and SPSIM. For both algorithms tested (SP-PSO-CIQ and SP-IDE-PSO-CIQ), the percentage loss of the HPSI index values was in the range of 0.14–4.66% and for SPSIM it was in the range of 0.02–0.74%. Such changes can be considered minor changes in quality.

**Figure 5:**
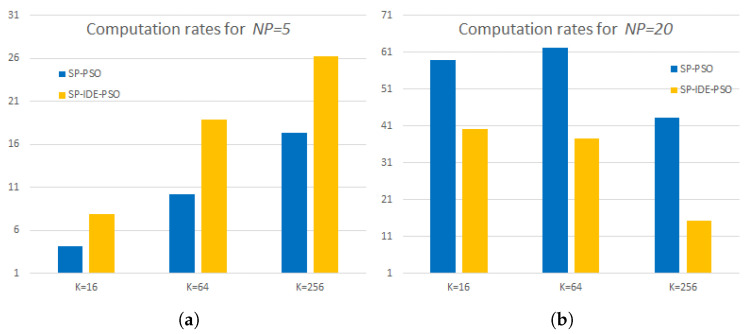
Computation rates of both superpixel CIQ methods for two swarm sizes. (**a**) NP=5. (**b**) NP=20.

An important criterion for evaluating superpixel CIQ algorithms is the computation rate, defined as the ratio of the computation time of the tested algorithm in the pixel version to its computation time in the superpixel version. Figure 5 shows the computation rates of the tested superpixel versus pixel methods for small (NP = 5) and large (NP = 20) swarms. For example, for 16 colors with swarm size NP = 5, the average computation times (in seconds) for the pixel versions of the SP-PSO-CIQ and SP-IDE-PSO-CIQ algorithms were 2.91 and 5.68, respectively, and for the superpixel version they were 0.69 and 0.72, respectively. For the small swarm, computation rates ranging from 4.2 to 17.3 were obtained for the SP-PSO-CIQ algorithm and for the SP-IDE-PSO-CIQ algorithm, the computation rates ranged from 7.8 to 26. For the large swarm, computation rates ranged from 43.3 to 62.1 for the SP-PSO-CIQ algorithm, and for the SP-IDE-PSO-CIQ algorithm these computation rates ranged from 15.4 to 40.1. The above average computation rates were obtained on 24 images from the Kodak collection, with a 10-fold quantization performed for each image. For a small swarm, higher computation rates were achieved by the superpixel version of the IDE-PSO algorithm, and for a large swarm, higher computation rates were achieved by the super-pixel version of the PSO algorithm.

## 4. Conclusions

In this paper, two particle-swarm-optimization-based algorithms (PSO, IDE-PSO) are considered. Both algorithms have been applied to the important task of digital color image processing, i.e., in color image quantization (CIQ).This is the first time the IDE-PSO algorithm has been used in this field. Additionally, superpixel versions of both algorithms were considered, in which the number of superpixels used for adaptive palette generation depends on the palette size. The use of the superpixel approach is part of the novelty of the article. The experimental results show that the use of the IDE-PSO algorithm for CIQ yields better image quality than the classic PSO version, although over a longer time. The study was conducted on a large set of benchmark natural images. To evaluate the results obtained, ten quality indices (pixel-based, patch-based, and superpixel-based) were used, as well as a computation rate that determines the acceleration of calculations. Regardless of the swarm size, the advantage of the IDE-PSO-CIQ algorithms in both pixel and superpixel implementations is noticeable. The introduction of superpixel versions has made it possible to achieve a significant acceleration of PSO algorithm calculations with a small loss of image quality. The computing rates obtained are in the range of 4.2–62.1 with a quality loss of a few percent at most. In future research, we can improve the results using a different, more efficient superpixel decomposition method instead of the classic SLIC algorithm. This research will be important for all those who want a fast and high-quality color reduction method in computer vision tasks.

## Figures and Tables

**Table 1:** Comparison of MSE values between SOM, GCMA, PSO-CIQ, and IDE-PSO-CIQ.

Image	K	SOM	GCMA	PSO-CIQ	IDE-PSO-CIQ
Lenna	16	235.6 ± 0.490	332	210.203 ± 1.487	210.408 ± 1.228
	32	126.400 ± 1.200	179	119.167 ± 0.449	118.478 ± 0.624
	64	74.700 ± 0.458	113	77.846 ± 16.132	72.184 ± 0.256
Peppers	16	425.600 ± 13.162	471	399.63 ± 2.636	372.813 ± 2.824
	32	244.500 ± 3.854	263	232.046 ± 2.295	219.593 ± 1.122
	64	141.600 ± 0.917	148	137.322 ± 3.376	130.338 ± 0.978
Jet	16	121.700 ± 0.458	199	122.867 ± 2.0837	121.886 ± 1.968
	32	65.000 ± 0.000	96	71.564 ± 6.089	63.769 ± 1.241
	64	38.100 ± 0.539	54	56.339 ± 11.15	38.084 ± 0.909
Mandrill	16	629.000 ± 0.775	606	630.975 ± 2.059	631.102 ± 2.236
	32	373.600 ± 0.490	348	375.933 ± 3.42	373.592 ± 0.655
	64	234.000 ± 0.000	213	237.331 ± 2.015	234.93 ± 0.372

**Table 2:** Multi-index quality evaluation: PSO-CIQ vs. IDE-PSO-CIQ.

Image	K	Alg.	PSNR	DE	FSIM	SFF	ICID	DSCSI	HPSI	MDSIS	SPSIM
Lenna	16	PSO-CIQ	**29.67**	**6.111**	0.9480	0.9810	0.0906	**0.7673**	0.8423	0.3096	0.9620
		IDE-PSO-CIQ	29.65	6.145	**0.9502**	**0.9817**	**0.0901**	0.7669	**0.8473**	**0.3060**	**0.9631**
	32	PSO-CIQ	32.05	4.915	0.9767	0.9900	0.0478	0.8619	0.9224	0.2525	0.9832
		IDE-PSO-CIQ	**32.17**	**4.853**	**0.9772**	**0.9904**	**0.0466**	**0.8665**	**0.9253**	**0.2492**	**0.9838**
	64	PSO-CIQ	34.10	4.066	0.9890	0.9944	0.0279	0.9166	0.9568	0.2097	0.9920
		IDE-PSO-CIQ	**34.32**	**3.966**	**0.9899**	**0.9946**	**0.0262**	**0.9214**	**0.9587**	**0.2064**	**0.9926**
Peppers	16	PSO-CIQ	27.19	**8.109**	0.9139	0.9688	0.1498	0.6337	0.7416	0.3472	0.9330
		IDE-PSO-CIQ	**27.20**	8.111	**0.9144**	**0.9692**	**0.1482**	**0.6352**	**0.7429**	**0.3464**	**0.9339**
	32	PSO-CIQ	29.44	6.517	0.9553	0.9821	0.0838	0.7625	0.8494	0.2897	0.9666
		IDE-PSO-CIQ	**29.49**	**6.477**	**0.9557**	**0.9824**	**0.0827**	**0.7632**	**0.8501**	**0.2895**	**0.9668**
	64	PSO-CIQ	31.64	5.093	0.9747	0.9886	0.0517	0.8414	0.9074	0.2538	0.9811
		IDE-PSO-CIQ	**31.75**	**5.024**	**0.9760**	**0.9891**	**0.0499**	**0.8464**	**0.9094**	**0.2502**	**0.9824**
Jet	16	PSO-CIQ	32.01	3.498	0.9650	0.9860	0.0417	0.8157	0.8736	0.2743	0.9807
		IDE-PSO-CIQ	**32.04**	**3.486**	**0.9669**	**0.9860**	**0.0395**	**0.8208**	**0.8756**	**0.2704**	**0.9825**
	32	PSO-CIQ	34.68	2.909	0.9837	0.9924	0.0245	0.8850	0.9325	0.2210	0.9915
		IDE-PSO-CIQ	**34.86**	**2.848**	**0.9847**	**0.9926**	**0.0225**	**0.8890**	**0.9350**	**0.2168**	**0.9929**
	64	PSO-CIQ	36.69	2.550	0.9919	0.9951	0.0170	0.9219	0.9565	0.1848	0.9955
		IDE-PSO-CIQ	**37.10**	**2.427**	**0.9929**	**0.9953**	**0.0150**	**0.9264**	**0.9600**	**0.1800**	**0.9965**
Mandrill	16	PSO-CIQ	**24.91**	**11.338**	0.9376	**0.9674**	0.1915	**0.6532**	0.7505	0.3377	0.9526
		IDE-PSO-CIQ	24.90	11.445	**0.9384**	0.9666	**0.1910**	0.6499	**0.7550**	**0.3363**	**0.9537**
	32	PSO-CIQ	27.15	9.370	**0.9670**	**0.9791**	0.1251	**0.7635**	**0.8386**	**0.2818**	**0.9744**
		IDE-PSO-CIQ	**27.18**	**9.365**	0.9669	0.9789	**0.1251**	0.7596	0.8380	0.2824	0.9743
	64	PSO-CIQ	29.16	7.587	0.9810	0.9873	0.0773	0.8439	0.8979	0.2421	0.9858
		IDE-PSO-CIQ	**29.19**	**7.572**	**0.9814**	**0.9873**	**0.0763**	**0.8442**	**0.8997**	**0.2415**	**0.9859**

**Table 3:** Comparison of pixel and superpixel versions of PSO-CIQ and IDE-PSO-CIQ (K=16).

K = 16	MSE	PSNR	DE	FSIM	SFF	ICID	DSCSI	HPSI	MDSIS	SPSIM
NP=5										
PSO-CIQ	89.36	28.99	5.955	0.9385	0.9737	0.1383	0.7047	0.7740	0.3123	0.9582
IDE-PSO-CIQ	**83.11**	**29.37**	**5.645**	**0.9431**	**0.9760**	**0.1258**	**0.7196**	**0.7881**	**0.3047**	**0.9622**
SP-PSO-CIQ	108.07	28.10	6.370	0.9251	0.9703	0.1562	0.6691	0.7477	0.3324	0.9532
SP-IDE-PSO-CIQ	93.09	28.78	5.895	0.9334	0.9745	0.1367	0.6981	0.7721	0.3203	0.9596
NP=20										
PSO-CIQ	67.56	30.18	5.337	0.9502	0.9794	0.1156	0.7443	0.8090	0.2943	0.9663
IDE-PSO-CIQ	**67.39**	**30.21**	**5.299**	**0.9508**	**0.9795**	**0.1137**	**0.7462**	**0.8102**	**0.2934**	**0.9669**
SP-PSO-CIQ	93.22	28.78	5.911	0.9329	0.9745	0.1375	0.6970	0.7713	0.3209	0.9591
SP-IDE-PSO-CIQ	91.89	28.83	5.845	0.9339	0.9747	0.1351	0.7004	0.7742	0.3196	0.9601

**Table 4:** Comparison of pixel and superpixel versions of PSO-CIQ and IDE-PSO-CIQ (K=64).

K = 64	MSE	PSNR	DE	FSIM	SFF	ICID	DSCSI	HPSI	MDSIS	SPSIM
NP=5										
PSO-CIQ	30.24	33.72	3.699	0.9789	0.9907	0.0540	0.8615	0.9088	0.2336	0.9861
IDE-PSO-CIQ	**22.17**	**35.09**	**3.230**	**0.9852**	**0.9928**	**0.0391**	**0.8924**	**0.9330**	**0.2117**	**0.9909**
SP-PSO-CIQ	34.99	33.05	3.904	0.9767	0.9895	0.0579	0.8494	0.9032	0.2438	0.9854
SP-IDE-PSO-CIQ	28.23	33.98	3.593	0.9823	0.9916	0.0466	0.8772	0.9231	0.2260	0.9890
NP=20										
PSO-CIQ	20.08	35.51	3.236	0.9877	0.9938	0.0384	0.9042	0.9403	0.2038	0.9917
IDE-PSO-CIQ	**19.00**	**35.79**	**3.094**	**0.9887**	**0.9939**	**0.0348**	**0.9086**	**0.9439**	**0.1989**	**0.9927**
SP-PSO-CIQ	28.61	33.92	3.664	0.9819	0.9917	0.0482	0.8762	0.9222	0.2269	0.9885
SP-IDE-PSO-CIQ	27.32	34.14	3.542	0.9831	0.9919	0.0450	0.8815	0.9260	0.2232	0.9895

**Table 5:** Comparison of pixel and superpixel versions of PSO-CIQ and IDE-PSO-CIQ (K=256).

K = 256	MSE	PSNR	De76	FSIM	SFF	ICID	DSCSI	HPSI	MDSIS	SPSIM
NP=5										
PSO-CIQ	11.18	38.10	2.388	0.9939	0.9965	0.0196	0.9428	0.9682	0.1716	0.9961
IDE-PSO-CIQ	**9.43**	**38.87**	**2.176**	0.9950	0.9968	**0.0156**	0.9511	0.9732	0.1625	0.9971
SP-PSO-CIQ	12.47	37.57	2.478	0.9936	0.9961	0.0203	0.9403	0.9669	0.1765	0.9959
SP-IDE-PSO-CIQ	9.56	38.70	2.259	**0.9955**	**0.9971**	0.0156	**0.9560**	**0.9748**	**0.1612**	**0.9972**
NP=20										
PSO-CIQ	7.17	40.00	2.094	0.9967	0.9978	0.0138	0.9647	0.9800	0.1490	0.9978
IDE-PSO-CIQ	**6.60**	**40.41**	**1.974**	**0.9971**	**0.9979**	**0.0120**	**0.9670**	**0.9816**	**0.1445**	**0.9982**
SP-PSO-CIQ	9.86	38.55	2.339	0.9954	0.9972	0.0166	0.9558	0.9744	0.1621	0.9970
SP-IDE-PSO-CIQ	9.31	38.82	2.247	0.9957	0.9972	0.0153	0.9576	0.9757	0.1593	0.9973

## Data Availability

The Kodak image dataset is available at: http://r0k.us/graphics/kodak/ (accessed on 2 December 2022). Other data is not available.

## Abbreviations

In this manuscript, the following abbreviations are used:

CIQ	Color Image Quantization
RGB	Red, Green, Blue
KM	K-Means
FCM	Fuzzy C-Means
SOM	Self-Organizing Map
GCMA	Genetic C-Means Algorithm
PSO	Particle Swarm Optimization
PSO-CIQ	Particle Swarm Optimization for CIQ
IDE-PSO-CIQ	Individual Difference Evolution PSO for CIQ
SLIC	Simple Linear Iterative Clustering
CIELab	Lab color space defined by CIE
PSNR	Peak Signal-to-Noise Ratio
FSIM	Feature SIMilarity index (color version)
SFF	Sparse Feature Fidelity
MDSI	Mean Deviation Similarity Index
ICID	Improved Color Image Difference
DSCSI	Directional Statistics Color Similarity Index
HPSI	Haar wavelet Perceptual Similarity index
SPSIM	SuperPixel SIMilarity index
SP-PSO-CIQ	SuperPixel version of PSO-CIQ
SP-IDE-PSO-CIQ	SuperPixel version of IDE-PSO-CIQ
